# Rotavirus Vaccination of Infants Delayed and Limited within the National Immunization Programme in the Netherlands: An Opportunity Lost

**DOI:** 10.3390/vaccines9020144

**Published:** 2021-02-10

**Authors:** Florian Zeevat, Evgeni Dvortsin, Abrham Wondimu, Jan C. Wilschut, Cornelis Boersma, Maarten J. Postma

**Affiliations:** 1 Department of Health Sciences, University Medical Centre, University of Groningen, 9713 AV Groningen, The Netherlands; a.w.dagne@rug.nl (A.W.); cornelisboersma@health-ecore.com (C.B.); m.j.postma@rug.nl (M.J.P.); 2 Asc Academics, 9713 GH Groningen, The Netherlands; evgeni@ascacademics.com; 3 Department of Pharmaceutics, School of Pharmacy, College of Medicine and Health Sciences, University of Gondar, Gondar P.O. Box 196, Ethiopia; 4 Department of Medical Microbiology and Infection Prevention, University Medical Center Groningen, University of Groningen, 9713 AV Groningen, The Netherlands; jcwilschut@gmail.com; 5 Faculty of Management Sciences, Open University, 6419 AT Heerlen, The Netherlands; 6 Department of Economics, Econometrics & Finance, University of Groningen, Faculty of Economics & Business, 9700 AV Groningen, The Netherlands

**Keywords:** National Immunization Programme, vaccination, public health, rotavirus vaccine, costs and effects

## Abstract

In this study, we estimated the benefits of rotavirus vaccination for infants had the rotavirus vaccine been introduced in the Netherlands as of its market authorization in 2006. An age-structured, deterministic cohort model was developed to simulate different birth cohorts over a period of 15 years from 2006 until 2021, comparing both universal and targeted high-risk group vaccination to no vaccination. Different scenarios for the duration of protection (5 or 7 years) and herd immunity (only for universal vaccination) were analyzed. All birth cohorts together included 2.6 million infants, of which 7.9% were high-risk individuals, and an additional 13.2 million children between 1–15 years born prior to the first cohort in 2006. The costs and health outcomes associated with rotavirus vaccination were calculated per model scenario and discounted at 4% and 1.5%, respectively. Our analysis reveals that, had rotavirus vaccination been implemented in 2006, it would have prevented 356,800 (51% decrease) and 32,200 (5% decrease) cases of rotavirus gastroenteritis after universal and targeted vaccination, respectively. Over the last 15 years, this would have led to significant avoided costs and quality-adjusted life year losses for either vaccination strategy with the most favorable outcomes for universal vaccination. Clearly, an opportunity has been lost.

## 1. Introduction

In the Netherlands, the National Immunization Programme (NIP) incorporates vaccines that protect against 12 infectious diseases in children [1], leading to a major decrease in childhood mortality and a consequential increase in life expectancy [2]. Not every available vaccine against infectious diseases is included in the NIP. One vaccine not included is the rotavirus vaccine marketed as GSK’s Rotarix^®^ or MSD’s RotaTeq^®^, both of which were granted market authorization by the European Medicines Agency (EMA) in 2006 [3,4]. The vaccine protects against rotavirus gastroenteritis (RVGE), which is the leading cause of dehydration in young infants and may lead to hospitalization and even death [5,6]. After long delays in the assessment, in 2017 the Netherlands Health Council published a recommendation indicating that both universal and targeted rotavirus vaccination would lead to major benefits [7]. Subsequently, in 2019, the inclusion of the rotavirus vaccination in the Dutch NIP was decided upon, but for high-risk children only (targeted vaccination) [8]. However, due to difficulties in the implementation process and a possibly lower vaccine effectiveness among high-risk infants, rotavirus vaccination was not pursued, and in early 2020, new advice from the Health Council was requested [9]. 

The above decision-making process illustrates that from the moment a vaccine receives market authorization from the EMA, it may take a significant period of time before the vaccine is included in the Dutch NIP. The final decision is taken by the Ministry of Health, Welfare, and Sports, but the minister first asks advice from the Health Council. The Health Council makes an assessment as to whether the vaccine meets a total of seven crucial criteria [10]: the seriousness of the disease burden, the extent of the disease burden, the effectiveness of the vaccine, the safety of the vaccine, the acceptability of the vaccination, the efficiency of the vaccination, and the priority of vaccination [11]. After a positive recommendation by the Health Council, and after the minister has decided to include the vaccination in the NIP, the National Institute for Public Health and the Environment is responsible for the implementation of the vaccination by taking care of the invitations to children and their parents and by tracking the vaccination status of every child, as well as purchasing, storing, and distributing the vaccine. 

As indicated above, for the rotavirus vaccine it took more than 10 years before formal advice was given by the Health Council in the Netherlands. However, despite the positive advice of the Health Council, universal or targeted rotavirus vaccination is still not included in the NIP. In contrast to the Netherlands, universal vaccination for RVGE was introduced in Belgium in 2006. This has resulted in a 78% decrease in hospitalizations of children aged <5 years after two years of vaccination, and a 63% decrease in rotavirus infections for individuals aged 10 years and older [12]. Within this perspective, it can be assumed that the delay between EMA approval and the inclusion of rotavirus vaccination in the Dutch NIP has led to a significant loss of health benefits [13]. Indeed, multiple infections and consequent hospitalizations due to RVGE could have been prevented by an early introduction of universal rotavirus vaccination. In this study, we aim to calculate the hypothetical benefits of immediate introduction of the rotavirus vaccine for all infants or just high-risk infants in the Netherlands in 2006, versus the actual situation of no introduction until recently. Thus, this study quantifies the opportunity that has been lost. 

## 2. Materials and Methods

### 2.1. Rotavirus Model

**Figure 1:**
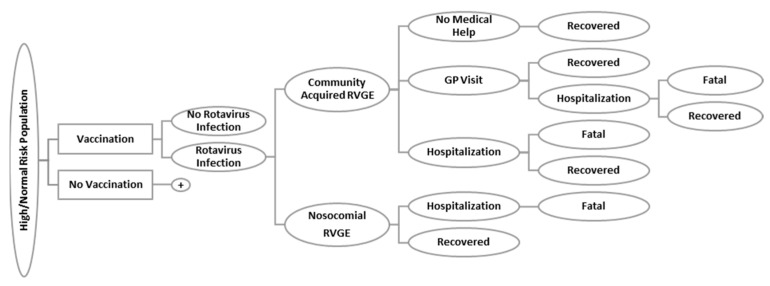
Schematic structure of the age-structured deterministic cohort model: the covered and non-covered patients (vaccination/no-vaccination) are both at risk of acquiring rotavirus infection in community or hospital (nosocomial). The infection can lead to complications in which the patient will stay at home (no medical help), visit the GP, or become hospitalized. The probability of dying because of rotavirus infection is only included within the cases who are hospitalized. The “no vaccination” arm is the same as the “vaccination” arm (+), except that different probabilities apply. The population considered can be at normal or high risk for rotavirus infection. GP, general practitioner; RVGE, rotavirus gastroenteritis.

A cost-effectiveness model was adapted to calculate the benefits and costs of the inclusion of the rotavirus vaccine in the Dutch NIP over the period from 1 July 2006 to 30 June 2021 (fifteen years), assuming that the rotavirus vaccine will be implemented in 2021. An age-structured, deterministic cohort model was used, based on a previous model that was developed to estimate the cost-effectiveness of vaccination with RotaTeq^®^ [13,14]. The model comprised of 5 health states (Figure 1): (1) community-acquired (CA) RVGE not requiring any medical services, (2) CA RVGE treated by a general practitioner (GP) but not requiring hospitalization, (3) non-fatal CA RVGE requiring hospitalization, (4) non-fatal hospital-acquired (nosocomial) RVGE case, and (5) fatal CA or nosocomial RVGE. These health states were modelled for different age-groups: 0–2 months, 2–4 months, 4–6 months, 1–2 years, 2–3 years, 3–4 years, 4–5 years, 5–9 years, 9–15 years, and 15 years and older. Calculations were performed using Microsoft Excel^®^ 2016. 

The rotavirus vaccine (Rotateq^®^) was offered annually to newborns and was administered at 2, 4, and 6 months of age. As we do not expect our results to vastly differ for Rotarix^®^, we refer in our analysis below to “rotavirus vaccine” in general which may be conceived as reflecting either vaccine. Both universal and targeted vaccination were investigated, reflecting, respectively, the Belgian approach from 2006 and the proposed Dutch strategy expected to be implemented soon. The effects of universal or targeted vaccination were modelled as a reduction in RVGE and the concomitant direct healthcare costs and quality-adjusted life-years (QALY) losses avoided for a vaccinated population compared with a non-vaccinated population.

Specifically, the targeted vaccination strategy restricts vaccination to high-risk infants only, rather than vaccination of the whole birth cohort against RVGE [15]. Infants at risk for RVGE are those with prematurity of <36 weeks gestational age, low birth weight, and/or severe congenital malformations expected to last longer than 12 months and requiring inpatient paediatric care, associated with increased healthcare needs (i.e., hospitalization costs) and high mortality risk [13].

The birth cohorts for each year from 2006 through 2020 were included in the model [16], of which 7.9% were eligible for rotavirus vaccination (high-risk individuals). In order to include the potential benefits for the infants vaccinated within the study period after June 2021, we included a lifetime horizon for every birth cohort between 2006 and 2021. 

### 2.2. Probabilities of Incidence, General Practitioner Visits, Hospitalizations, and Mortality of Rotavirus Infection

Input data was based on a previously published cost-effectiveness model of rotavirus vaccination in the Netherlands [17]. This study obtained incidence estimates of the health states (CA RVGE, GP visits, hospitalizations, and nosocomial RVGE) by risk category (normal/high-risk) from three observational studies (Sensor cohort study; NIVEL; RoHo study), and updated the input parameters by using rotavirus surveillance data of the years 2013–2017 [17].

Incidence rates for those with no need for medical help, GP visits, hospitalizations for CA RVGE-cases, hospitalizations for nosocomial cases, and mortality (Table 1), include different age distributions for RVGE incidence, hospitalizations, and mortality (Table 2) [13,14]. Different mortality rates were applied for high-risk hospitalized children versus the rest of the general annual birth cohort, based on the assumption that children would only die from rotavirus infection after hospitalization. 

### 2.3. Costs

Rotavirus-related costs were divided into three categories: direct costs for healthcare utilization as well as costs associated with complications (hospitalization, GP visits, prescription, and laboratory costs), direct non-healthcare costs such as additional diapers and travel costs, and indirect non-healthcare costs such as productivity losses for the caregiver (Table 3) [13,18].

Hospitalization costs were based on a regular ward room stay per day, taken from a previously published study [13] and included costs for tertiary hospital care, general hospital care, intensive care, and emergency room visits. Additional costs for contact isolation, ambulance transport, staff, consumables, overheads, diagnostic tests, and pharmaceuticals, were also included [13]. Costs for diagnosis and therapeutic intervention were only included for nosocomial hospitalizations, and only those involving microbiology testing, blood chemistry or haematology, and ultrasound or X-ray imaging [13]. Mean costs for GP visits were derived based on a split of 90% practice visits to 10% home visits, including 0.97 additional phone calls per registered GP visit [19]. Total costs for patients receiving an antibiotic, analgesic, or ORS prescription after a GP consultation, associated with medication costs and pharmacy prescriptions were taken from a Dutch cost-effectiveness analysis [13]. 

Travel costs to the GP and hospital were calculated in line with the Dutch Manual for Costing Research [18], assuming that 25% of the GP visits and 50% of the hospital visits use either car or public transport [14]. Other indirect costs, such as costs for two additional diapers per day, were included for all children up to 1 year old and 50% of the children aged 1–4 years old [20]. Productivity losses were only included for the informal caregiver, including the average hourly labor costs for both men and women and including a work elasticity of 0.8 [18]. Duration of illness was assumed to be 4.9 days for cases requiring no medical help, 7.1 days for cases requiring a GP visit, and 7.7 days for cases requiring hospitalization [14]. Similar to other rotavirus cost-effectiveness analyses, adverse events following rotavirus vaccination were not included, as they are considered absent or extremely mild [13,20]. Vaccination costs were not included in this analysis. All costs were annually adjusted to the cost-year, depending on the year of the birth cohort from 2006 through 2020 (using the consumer price index from The Netherlands’ Central Bureau of Statistics) [16]. 

### 2.4. Quality-Adjusted Life-Years

QALYs were obtained from a Canadian study in which caregivers evaluated health-related quality of life for their children and themselves [21] and are used to reflect the quality of life losses due to RVGE among children and their informal caregivers (Table 3). QALY loss due to morbidity is the product of the difference in utility weight between RVGE disease and the fully healthy state (1.0), and the length of duration in that state during an RVGE episode. QALY losses due to RVGE mortality were based on life expectancy estimates and the modelled mortality incidence [13]. 

### 2.5. Vaccine Efficacy, Coverage, and Herd Immunity 

We applied six scenarios in which we include a 5- or 7-year duration of protection after vaccination with the rotavirus vaccine, with or without herd immunity (Table 4). The rotavirus vaccination scheme consisted of administration of a dose at two months, four months, and six months after birth. Vaccine efficacy was derived from the European cohort of the Rotavirus Efficacy and Safety Trial (REST) [22], stating that the efficacy of the first full season after vaccination should be multiplied by the following dose-specific scale factors: first dose by 1/1.073^2, second dose by 1/1.073, and third dose by 1.0 (Table 5). Efficacy during the third to the fifth season was calculated as a linear decline equal to the reduction between the first and second season, as was performed in a previously published cost-effectiveness study [17]. The efficacy during the sixth and seventh season was obtained from a surveillance study that assessed the vaccine effectiveness in children over the period 2012–2013 (Table 5) [23]. 

The lowest annual vaccine coverage rate of infants in the NIP, for the different vaccines included, was observed in 2016 and ranged from 92% to 93% [24]. Therefore, average vaccination coverage of 92.5% was used for both universal vaccination and targeted vaccination. We applied scenarios 3–6 to universal vaccination only, as herd protection is only effective with universal vaccination. Herd protection was assumed after the third year of vaccination (after 2009), as we expect that three years after vaccine introduction the vaccine coverage rate will be high enough for herd immunity effects. We included the indirect effects through June 2024, as we assumed the rotavirus vaccine will be implemented in June 2021 and that it takes three years to achieve herd immunity. In the last two scenarios (scenarios 5 and 6), we assumed herd protection for the population outside those vaccinated, including infants and children born 15 years prior to the hypothetically vaccinated cohorts. Herd immunity effects were based on a recently published cost-effectiveness and risk–benefit analysis of rotavirus vaccination (Table 6) [17]. 

### 2.6. Outcomes

For every scenario, the RVGE incidence numbers, complications, and potential deaths were calculated. The total health effects (QALY losses) and costs (direct and societal) of rotavirus vaccination were calculated, relative to the RVGE incidence for each scenario. In addition, the average costs and QALYs per year and per born child were calculated. The standardized difference between no vaccination and vaccination (universal/targeted) was calculated as Cohen’s effect size, in terms of costs and QALYs per year [26]. Health effects and costs after 30 June 2020, were discounted according to the Dutch guidelines for costing studies in healthcare at 1.5% and 4%, respectively. 

## 3. Results

### 3.1. Incremental Effects 

According to our model, universal vaccination with a 5-year duration of protection, but without herd immunity (scenario 1) would have prevented an estimated 420,838 rotavirus infections representing a 46% decrease in cases, while a targeted vaccination programme would have prevented 35,161 rotavirus infections representing a 4% decrease. Assuming a 7-year duration of protection (scenario 2), vaccination would have prevented 445,906 cases (49% decrease) or 37,369 (4% decrease) after universal vaccination or targeted vaccination, respectively. On average, targeted vaccination would have resulted in a reduction of 6274 GP visits (4% decrease), 6198 CA hospitalizations (10% decrease), and 2466 nosocomial hospitalizations (28% decrease) for both a 5- or a 7-year duration of vaccine-induced protection. The number of deaths avoided was comparable for either vaccination strategy at 32 deaths using a 5-years duration of protection and 33 deaths using a 7-years duration of protection. Universal vaccination was shown to be very effective in preventing healthcare visits. Indeed, on average 79,219 GP visits (49% decrease), 50,859 CA hospitalizations (80% decrease), and 4711 nosocomial hospitalizations (54% decrease) were avoided compared to no vaccination.

Scenarios 3–6 were shown to be very effective among the infants as this scenario benefits from the direct as well as the herd effects of rotavirus vaccination. Scenarios 3 and 4 would have prevented 434,305 and 459,374 RVGE cases, respectively, and thus, approximately 3% more cases would have been prevented, compared to assuming no herd protection. Ergo, more GP visits and hospitalizations would have been prevented due to herd protection of the unvaccinated. The number of deaths avoided stayed the same: 32 cases with a 5-year duration of protection and 33 cases with a 7-year duration of protection. Scenario 5 and 6 included an additional 13 million children between 1 and 15 years of age. Scenario 5 would have prevented 499,249 RVGE cases (44% decrease), equivalent to an additional 64,944 cases avoided (38% decrease), compared to scenario 3. The number of hospitalizations and deaths averted was almost the same for scenarios 3 and 5. However, a difference was found in the number of mild RVGE cases (GP visit and no medical help). 

The cases that were prevented in the first two scenarios translated in 1091 or 1129 QALY losses avoided for universal vaccination and 531 or 544 QALY losses avoided for targeted vaccination against RVGE assuming a 5- or 7-year duration of protection, respectively (Table 7). When utility losses for caregivers were included, these results were even stronger: at least 2819 QALY losses avoided for universal vaccination and 1050 QALY losses avoided for targeted vaccination. The inclusion of herd protection could have led to 1109 (scenario 3) or 1148 QALY losses (scenario 4) avoided, which represents a 2% increase in prevented QALY losses for either duration of protection. Inclusion of a greater cohort in the model (scenario 5–6) resulted in 1186 QALY losses (scenario 5) and 1224 QALY losses (scenario 6) avoided. 

The average avoided QALY loss per birth was calculated at 0.0004 for all birth cohorts over the past 15 years (Table 8). The inclusion of the caregivers resulted in an avoided QALY loss of approximately 0.0011 per birth. The average annual avoided QALY loss for the included birth cohorts from 2006 until 2021 was 71.7 or 179.3 without or with the inclusion of QALY losses for the caregivers, respectively. The effect sizes of the QALY loss were large, according to benchmarks (d = 0.5–0.8: medium effect size; d > 0.8: large effect size).

### 3.2. Incremental Costs

In addition to the avoided QALY losses described above, universal vaccination and targeted rotavirus vaccination would have avoided at least EUR 138.0 million and EUR 23.1 million direct costs, respectively, compared to no vaccination (Table 7). Applying a societal perspective, we found that universal vaccination was the most favorable vaccination strategy, avoiding more than 74% (EUR 198.6 million) of the total costs due to RVGE, compared to a 12% reduction (EUR 31.1 million) following targeted vaccination. Scenario 5 and 6 resulted in societal cost-savings of minimally EUR 204.5 million, which corresponds to a cost reduction of 75%.

The average costs avoided were estimated at EUR 53.38 or EUR 77.00 per birth over the past 15 years from a third-party payer or societal perspective, respectively (Table 8). The targeted strategy avoided on average EUR 8.81 third-party payer costs and EUR 12.05 societal costs per newborn child. This translates to costs that could have been avoided on an annual basis of EUR 8,799,706 (direct costs) and EUR 12,694,494 (societal costs) for universal vaccination. For targeted vaccination, the annual costs avoided were estimated at EUR 1,451,822 (direct costs) and EUR 1,986,633 (societal costs). All effects sizes for universal and targeted vaccination were large and medium, respectively.

## 4. Discussion

According to our model, implementation of the rotavirus vaccination would have prevented a significant number of RVGE cases and costs among young children in the Netherlands over the period 2006 to 2021. This holds, especially true for universal vaccination. Indeed, it was estimated that more than 50% of cases would have been prevented had universal vaccination been implemented. Prevention of infectious diseases (such as RVGE) will improve health outcomes and reduce disease-related costs. In our analysis, the annual direct healthcare costs that could have been avoided were estimated at approximately EUR 8.6 million. This is comparable with the healthcare savings of approximately GBP 7.5 (EUR 10.5) million after the first year that rotavirus vaccination was implemented in the NIP in the United Kingdom [27]. From societal perspective, an estimated EUR 13 million per year would have avoided by universal vaccination. For targeted vaccination, the annual costs avoided were estimated at EUR 2 million. Only a small difference between the two scenarios based on duration of protection and herd immunity was observed: a 1% difference between a 5- or 7-year duration of protection, and a 2.1% and 2.9% difference in herd immunity of the unvaccinated without and with children outside the vaccination cohort, respectively. 

The Netherlands is the first country that recommended and decided to implement targeted rotavirus vaccination in the NIP. Though currently discussed as a result of practical implementation challenges, the main reason for a positive recommendation was that targeted vaccination had been shown to be not only cost effective, but even cost saving [17]. Indeed, in the present study, we showed that vaccination of high-risk populations reduces mortality significantly. Almost the same number of deaths would have been prevented by the implementation of targeted vaccination compared to universal vaccination, since for the general birth cohort the mortality rate is very low (0.005%), compared to that among high-risk infants (0.81%) [10,11]. However, a chance in mortality will not have a significant impact on our cost outcomes, as productivity losses were only included for the caregivers and not for the patient in terms of lost future earnings due to premature death. Ergo, if these costs would have been included, a significantly higher difference in indirect costs will be observed between vaccination and no vaccination, as more cost could be prevented. Only a significant difference in QALY losses can be observed if the mortality rate is varied, as future QALY losses due to mortality were included in this analysis. 

Although targeted vaccination, compared to universal vaccination, would have prevented almost the same number of deaths against considerably lower costs, universal vaccination would have resulted in many more health benefits compared to targeted vaccination, and thus in prevention of considerably more healthcare and non-healthcare costs. Compared to the high reduction in overall RVGE incidence after universal vaccination (46% reduction), the incidence was hardly reduced after targeted vaccination (5% reduction). In our analysis, targeted vaccination was limited to health gains in terms of GP visits and hospitalizations (4% and 10–28%), compared to universal vaccination (49% and 54–80%). 

A major advantage of universal, compared to targeted, vaccination involves its significant effect on herd immunity. Due to herd immunity, universal vaccination will not only protect all the vaccinated infants but also the children outside the vaccination cohort. Therefore, many more RVGE cases will be avoided by universal vaccination compared to targeted vaccination; approximately 450,000 additional rotavirus infections in the period 2006 to 2021 were prevented in our analysis with universal vaccination. In addition, the vaccine effectiveness in hospitalized critically ill children could be lower than currently assumed [28], leading to a less effective programme with targeted vaccination compared to universal vaccination. Those who are at risk and/or too young to be effectively vaccinated will be protected by universal vaccination due to a significant level of herd immunity. Moreover, herd immunity effect is also seen in the adult and elderly population. In the UK, the overall incidence rate of acute gastroenteritis cases dropped by 12–16% in a high gastroenteritis season two years after the start of universal vaccination of infants [29]. Therefore, not implementing universal vaccination within the NIP seems like a lost opportunity from a health and economic perspective. 

In Europe, there is large variability between countries in the regulatory framework, decision making, and assessment of vaccines through the evaluation of cost-effectiveness studies [30]. European countries, such as the United Kingdom, Sweden, Norway, Italy, Germany, Belgium, and Austria already reimburse universal rotavirus vaccination [12]. Despite comparable cost-effectiveness outcomes for the rotavirus vaccination of EUR 72,021 and EUR 68,321 per QALY for the Netherlands and Belgium, respectively, rotavirus vaccination until now has not been implemented in the Netherlands. While the Netherlands had initially decided to implement targeted rotavirus vaccination in the NIP, the minister has now decided to await other advice from the Health Council. This is most likely related to the new vaccine effectiveness results among high-risk infants, and potential implementation challenges of the targeted vaccination related to the precise definition of high-risk infants, and the location and time of vaccination [31]. On the other hand, universal vaccination is much easier to implement in the NIP and will protect even more cases in absolute terms.

A limitation of our analysis relates to the inclusion of a constant probability rate for the incidence of RVGE. In our model, we included RVGE incidence rates estimated from the period 1996–2007 [14]. However, since 2013, the number of RVGE cases decreased by approximately 50% [32]. This reduction in incidence might be caused by the implementation of universal vaccination in neighbouring countries [32]. Ergo, with these dated incidence data, the total calculated cases prevented and cost avoided in our model could be overestimated. Another limitation relates to the costs used. Costs were obtained from literature, and the Dutch guidelines for costing studies and were calculated for different years by using the annual inflation rate. Thus, the costs can deviate from the actual prices [10,11,12]. 

In this study, we only described the costs that would have avoided by the introduction of rotavirus vaccination. Therefore, we did not include administration costs. Though these costs can be considered substantial for in particular vaccination of the entire annual birth cohort, universal vaccination may still be cost-effective at reduced vaccine costs [17]. In particular, cost-effectiveness analyses are generally based on official list prices for vaccines, but these prices often do not reflect the lower actual or net price paid for the vaccines for universal programmes. Moreover, as the Dutch government already established an infrastructure for universal vaccination strategies, we expect lower costs for the implementation process, compared to targeted vaccination. 

We included no adverse events caused by rotavirus vaccination, such as the increased risk of intussusception. However, intussusception has been considered previously as a serious side effect of rotavirus vaccination with an estimated risk of 1 per 50,000 children, of which 4.8% of cases are severe. For the current vaccines, an association between vaccination and intussusception has not been demonstrated, and in countries where universal rotavirus vaccination has been implemented, there has been no increase in cases of intussusception. However, prudently, the Dutch Health Council takes into account a potential annual number of maximally 4 cases of intussusception that might occur due to vaccination among 64 cases that will occur anyway [7]. Intussusception may resolve spontaneously, or otherwise can be readily treated. Nonetheless, related costs can be high (EUR 1423–6759) and may have a significant impact on QALY losses (0.0011–0.0037) and caregiver productivity losses [17]. For our results, the inclusion of intussusception will lead to a smaller amount of cost and QALY losses avoided. However, due to the very low incidence of intussusception, it may not have a considerable impact on the total costs and QALYs. 

Additionally, in addition to achieving control of RVGE, it is important to involve healthcare workers, especially workers in the hygiene sector and preventive medicine (public health professions), in the implementation process, given that healthcare workers have a serious role in vaccine promotion and informing the patient [33]. The investment in education of healthcare providers and the general public will increase the vaccination coverage and may achieve control of vaccine-preventable diseases. In particular, the coverage rate among healthcare workers, as they are high-risk for acquiring an infectious disease and infecting others, such as the susceptible in hospital (nosocomial infections). 

Before a new vaccine or vaccination strategy is implemented in the context of a NIP, the vaccine is subject to the European marketing authorization and national decision-making processes. These bodies require time to clinically assess the vaccine as well as to advice on reimbursement and purchasing price [34]. This study presents an alternative perspective by showing the loss of opportunity impact of the delayed decision on vaccination strategies. However, these results are not only important for the decision making in rotavirus vaccination, but also for new preventive interventions against other infectious diseases in the Netherlands.

## 5. Conclusions

Rotavirus vaccination, both targeted and universal, has been shown to be very effective in reducing the RVGE burden of disease among infants in the Netherlands, and could reduce the potentially high demand for hospital beds in winter and early spring. However, until now rotavirus vaccination has not been implemented in the Netherlands. The present study shows that this delay has led to a significant healthcare burden that could have been prevented.

While universal vaccination is generally associated with high costs, it should be noted that list prices of vaccines do not reflect the “true” cost of a vaccine in the NIP; significant price reductions following negotiations being not uncommon. Additionally, even when targeted rotavirus vaccination in the NIP is the preferred option in the Netherlands, it is important to keep in mind that there will be a high remaining burden of disease due to RVGE. This may involve disproportionately high costs compared to universal vaccination due to a more complicated implementation of targeted vaccination within the context of the NIP and including anticipated potential purchasing benefits for universal vaccination.

## Figures and Tables

**Table 1:** Total population and probabilities of incidence, general practitioner visits, hospitalizations, and mortality of rotavirus infection in the population.

Parameter	Value
Total Population	Depends on the Year of Entry (2006–2020) [16]
RVGE Incidence as Percentage of the Total Population [13]	
<1 year	9.9%
1 to 4 years	23.11%
5 to 9 years	5.02%
GP visits 0 to 1 years	2.10%
GP visits 1 to 4 years	4.32%
GP visits 5 to 14 years	0.29%
Hospitalization community-acquired RVGE	2.40%
Hospitalization nosocomial RVGE	0.27%
Mortality among hospitalized cases (high risk)	0.81%
Mortality among hospitalized cases (normal risk)	0.0049%

GP, general practitioner; RVGE, rotavirus gastroenteritis.

**Table 2:** Age distribution of rotavirus incidence, hospitalizations, and mortality for low and high-risk infants (source Bruijning-Verhagen et al. [13]).

Parameter	Age (years)									
	<1				1 to 2	2 to 3	3 to 4	4 to 5	5 to 9	10 to 14
	months									
	0 to 2	2 to 4	4 to 6	6 to 12						
RVGE incidence	2.0%	3.0%	4.0%	15.0%	37.3%	10.3%	7.3%	2.3%	57.0%	13.0%
Hospitalization–Normal risk										
Community-acquired	5.0%	5.0%	8.0%	28.0%	34.0%	9.0%	6.0%	2.0%	3.0%	0.0%
Nosocomial	11.0%	17.0%	11.0%	31.0%	15.0%	6.0%	4.0%	0.0%	2.0%	3.0%
Hospitalization–High risk										
Community-acquired	0.0%	9.2%	3.1%	21.4%	40.8%	13.3%	2.0%	4.1%	4.1%	2.0%
Nosocomial	29.7%	14.9%	6.9%	24.8%	16.8%	0.0%	5.0%	0.0%	2.0%	0.0%
Mortality										
Normal risk	14.3%	28.6%	0.0%	42.9%	14.3%	0.0%	0.0%	0.0%	0.0%	0.0%
High risk	14.3%	28.6%	0.0%	42.9%	14.3%	0.0%	0.0%	0.0%	0.0%	0.0%

**Table 3:** Dutch unit costs (EUR) and utilities used.

**COSTS**	**Unit Costs (2006**)		**Source**
	**Normal Risk**	**High Risk**	
GP visit	EUR 78.73		Dutch Costing Manual [18]
Hospitalization CA	EUR 1995.11	EUR 2334.80	Bruijning-Verhagen et al. [13]
Hospitalization nosocomial	EUR 1826.64	EUR 1949.33	
Additional diapers and clean baby wipes	EUR 0.32		Rozenbaum et al. [20]
Travel costs GP	EUR 0.74		Dutch Costing Manual [18]
Travel costs Hospital	EUR 2.44		
Productivity losses	EUR 30.00		
**UTILITY**	**QALY Loss**	**QALY Loss Informal Caregiver**	**Source**
Mild (no professional help)	0.0011	0.002	Brisson et al. [21]
Moderate (GP visit)	0.0022	0.004	
Severe (Hospitalization)	0.0034	0.004	

CA, community-acquired; GP, general practitioner; QALY; quality-adjusted life years.

**Table 4:** Scenarios 1 to 6.

Scenario	Herd Immunity	Duration of Protection	Vaccination Programme
1	NA	5 years	Universal; Targeted
2		7 years	Universal; Targeted
3	Herd protection of the non-covered children within the vaccination cohort	5 years	Universal
4		7 years	Universal
5	Herd protection of the non-covered children outside the vaccination cohort	5 years	Universal
6		7 years	Universal

NA, not applicable; Targeted, targeted vaccination; Universal, universal vaccination.

**Table 5:** Vaccine efficacy.

Vaccine Efficacy	Mild (No Medical Help)	Moderate (GP Visit)	Severe (Hospitalization)	Source
After first dose (at 2 months)	62.54%	62.54%	86.86%	Atkins et al. [25]
After second dose (at 4 months)	67.10%	67.10%	93.20%	Atkins et al. [25]
First season (after third dose, at 6 months)	72.00%	72%	100.00%	Vesikari et al. [22]
Second season	58.50%	58.50%	94.30%	Vesikari et al. [22]
Third season	45%	45%	89%	Calculated
Fourth, fifth season	31.5%	31.5%	82.9%	Calculated
Sixth, seventh season	69%	69%	69%	Payne et al. [23]

GP, general practitioner.

**Table 6:** Herd immunity for the population outside those vaccinated and the population born prior to the vaccinated cohort (unvaccinated).

	Age (years)							
	<1		1–2	2–3	3–4	4–5	6–10	10–14
	months							
	<3	3–12						
Unvaccinated, but within the otherwise vaccinated birth cohort	30%	25%	25%	25%	25%	25%	0%	0%
Unvaccinated outside the vaccinated birth cohorts	0%	0%	28%	28%	28%	28%	28%	28%

**Table 7:** Cohort- and time-horizon-dependent rotavirus incidence, general practitioner (GP) visits, hospitalizations, and mortality for universal, targeted, and no vaccination across different vaccine effectiveness scenarios.

Scenarios	Cohort *	RVGE Incidence	GP Visits	Hospitalizations, Community-Acquired	Hospitaliza-tions, Nosocomial	Mortality	QALY Loss	QALY Loss Incl Caregivers	Direct Costs	Societal Costs
Without herd immunity										
No vaccination	2,637,753	905,600	160,202	63,160	8750	92	2577	6309	EUR 181,735,458	EUR 267,975,295
Universal 5-years duration of protection		484,762	81,785	12,558	4059	60	1486	3490	EUR 43,712,402	EUR 69,351,960
7-years duration of protection		459,694	80,180	12,045	4020	59	1448	3393	EUR 42,420,563	EUR 67,160,891
Targeted 5-years duration of protection		870,439	153,999	57,004	6296	59	2046	5258	EUR 158,621,881	EUR 236,367,303
7-years duration of protection		868,230	153,857	56,921	6274	59	2033	5232	EUR 158,390,727	EUR 236,011,036
Herd Immunity **										
No vaccination	2,637,753	905,600	160,202	63,160	8750	92	2577	6309	EUR 181,735,458	EUR 267,975,295
Universal 5-years duration of protection		471,294	79,214	11,541	3956	60	1468	3432	EUR 40,797,308	EUR 65,077,486
7-years duration of protection		446,226	77,609	11,028	3917	59	1429	3335	EUR 39,505,470	EUR 62,886,417
Herd Immunity outside vaccination cohort ***										
No vaccination	15,844,177	1,137,542	175,238	63,331	8876	92	2849	6803	EUR 183,695,354	EUR 273,797,372
Universal 5-years duration of protection		638,293	90,040	11,664	4046	60	1664	3788	EUR 42,208,433	EUR 69,269,391
7-years duration of protection		613,225	88,436	11,151	4007	59	1625	3691	EUR 40,916,595	EUR 67,078,322

* A total of 7.9% of the population is high-risk. ** Herd immunity: herd protection of the non-covered children inside the vaccination cohort. *** Herd immunity outside vaccination cohort: herd protection of the non-covered children outside the vaccination cohort. GP, general practitioner; QALY, quality-adjusted life-years; Targeted, targeted vaccination; Universal, universal vaccination.

**Table 8:** Average avoided outcomes per year and per born child for universal and targeted vaccination.

		Without Herd Immunity		Herd Immunity **		Herd Immunity outside Vaccination Cohort ***	
		5 years Duration of Protection	7 years Duration of Protection	5 years Duration of Protection	7 years Duration of Protection	5 years Duration of Protection	7 years Duration of Protection
Average per birth							
Direct costs	Universal	EUR 52.33	EUR 52.82	EUR 53.43	EUR 53.92	EUR 53.64	EUR 54.13
	Targeted *	EUR 8.76	EUR 8.85				
Societal costs	Universal	EUR 75.30	EUR 76.13	EUR 76.92	EUR 77.75	EUR 77.54	EUR 78.37
	Targeted *	EUR 11.98	EUR 12.12				
QALY loss	Universal	0.0004	0.0004	0.0004	0.0004	0.0004	0.0005
	Targeted *	0.0002	0.0002				
QALY loss incl caregivers	Universal	0.0011	0.0011	0.0011	0.0011	0.0011	
	Targeted *	0.0004	0.0004				
Average per year							
Direct costs	Universal (95% CI)	EUR 8,626,441 (8,429,146–8,823,736)	EUR 8,707,180 (8,508,096–8,906,266)	EUR 8,808,634 (8,605,470–9,011,798)	EUR 8,889,374 (8,684,398–9,094,350)	EUR 8,842,933 (8,634,973–9,050,892)	EUR 8,923,672 (8,727,311–9,151,214)
	Effect size	5.63	5.70	5.78	5.84	5.42	5.47
	Targeted * (95% CI)	EUR 1,444,598 (1,411,433–1,477,764)	EUR 1,459,045 (1,425,572–1,492,519)				
	Effect size	0.73	0.74				
Societal costs	Universal (95% CI)	EUR 12,413,958 (12,135,100–12,692,817)	EUR 12,550,900 (12,268,803–12,832,998)	EUR 12,681,113 (12,394,536–129,67,690)	EUR 12,818,054 (12,528,198–13,107,912)	EUR 12,782,999 (12,480,807–13,085,190)	EUR 12,919,941 (12,614,469–13,225,413)
	Effect size	5.54	5.62	5.69	5.76	5.14	5.21
	Targeted * (95% CI)	EUR 1,975,499 (1,930,968–2,020,031)	EUR 1,997,766 (1,952,732–2,042,801)				
	Effect size	0.68	0.69				
QALY loss	Universal (95% CI)	68.2 (66.6–69.7)	70.6 (68.9–72.2)	69.3 (67.7–70.9)	71.7 (70.1–73.4)	74.1 (72.5–75.7)	76.5 (74.9–78.2)
	Effect size	2.74	2.86	2.80	2.92	1.79	1.86
	Targeted * (95% CI)	33.2 (32.4–33.9)	34.0 (33.2–34.8)				
	Effect size	1.21	1.24				
QALY loss incl caregivers	Universal (95% CI)	176.2 (172.1–180.2)	182.3 (178.0–186.5)	179.8 (175.7–184.0)	185.9 (181.6–190.2)	188.5 (184.4–192.7)	194.5 (190.3–198.9)
	Effect size	2.92	3.04	2.99	3.11	1.81	1.88
	Targeted * (95% CI)	65.6 (64.1–67.1)	67.3 (65.7–68.8)				
	Effect size	0.95	0.98				

* 7.9% of the population is high risk. ** Herd immunity: herd protection of the non-covered children inside the vaccination cohort. *** Herd immunity outside vaccination cohort: herd protection of the non-covered children outside the vaccination cohort. CI, confidence interval; QALY, quality-adjusted life-years; Targeted, targeted vaccination; Universal, universal vaccination.

## Data Availability

The data presented in this study are available within the article.
